# Self-regulated learning and academic engagement among university students: a latent mediation model of achievement emotions

**DOI:** 10.3389/fpsyg.2026.1822649

**Published:** 2026-06-11

**Authors:** Fengling Shi, Juan Li

**Affiliations:** School of Teacher Education, Heze University, Heze, China

**Keywords:** academic engagement, achievement emotions, latent mediation, self-regulated learning, structural equation modeling

## Abstract

Drawing on SRL theory and control–value theory, this study tested a latent parallel mediation model linking self-regulated learning (SRL) to academic engagement via positive and negative achievement emotions. Participants were 207 Chinese undergraduates who completed the MSLQ (SRL), AEQ-S (enjoyment/hope; anxiety/boredom), and the Student Engagement Scale. Latent SEM with MLR in Mplus showed excellent fit, χ^2^(183) = 200.48, *p* = 0.179; CFI = 0.991; TLI = 0.989; RMSEA = 0.021; SRMR = 0.040. SRL positively predicted positive emotions (β = 0.484, *p* < 0.001) and negatively predicted negative emotions (β = −0.456, *p* < 0.001); positive emotions were positively associated with engagement (β = 0.262, *p* = 0.001) and negative emotions were negatively associated with engagement (β = −0.297, *p* < 0.001). Both indirect effects were significant (β = 0.127, *p* = 0.006; β = 0.136, *p* = 0.001) and the direct SRL → engagement path remained significant (β = 0.332, *p* < 0.001), supporting partial mediation and indicating that SRL was associated with engagement partly through higher positive emotions and lower negative emotions.

## 1 Introduction

### 1.1 Academic engagement in higher education

Academic engagement has emerged as one of the most robust predictors of students' academic achievement, persistence, and wellbeing in higher education (Berhanu and Sewagegn, 2024). As universities worldwide confront increasing dropout rates (Gurski et al., 2025a), academic disengagement, and psychological distress among students (Gurski et al., 2025b), understanding the mechanisms that foster sustained engagement has become a central concern in educational psychology (Stoeber et al., 2011). Academic engagement refers to students' active involvement in learning activities, typically encompassing behavioral participation, cognitive investment, and emotional involvement (Birhan and Nurie, 2024). While engagement has been linked to academic success across diverse contexts (Furrer and Skinner, 2003; Reyes et al., 2012), less consensus exists regarding the psychological processes through which engagement develops and is sustained. In particular, the interplay between self-regulated learning and achievement emotions in shaping academic engagement remains underexplored at the latent construct level.

### 1.2 Self-regulated learning in higher education

Self-regulated learning (SRL) is widely recognized as a foundational competence in higher education (Molins and García, 2023). University students are expected to assume substantial responsibility for managing their own learning (Kondo et al., 2012), including setting goals, selecting strategies, monitoring progress, and adjusting behaviors in response to feedback. Theoretical models of SRL emphasize cyclical processes involving forethought, performance control, and self-reflection (Yang et al., 2025). These processes enable learners to actively shape their academic experiences rather than passively respond to instructional demands (University of Wroclaw, 2025). Empirical research consistently demonstrates that students with stronger SRL skills achieve higher academic performance (Broadbent, 2017), demonstrate greater persistence (Wang et al., 2025), and adapt more effectively to academic challenges (Sugiharto et al., 2025). However, much of this literature focuses on cognitive and metacognitive outcomes (Banihashem et al., 2025), while comparatively fewer studies investigate how SRL shapes students' emotional experiences (Järvenoja and Järvelä, 2009) and engagement (Ng et al., 2024).

#### 1.2.1 Conceptualization and measurement of self-regulated learning

The Motivated Strategies for Learning Questionnaire (MSLQ) operationalizes SRL as a multidimensional construct encompassing cognitive strategies (Duncan and McKeachie, 2005), metacognitive regulation (Khosrozadh et al., 2025), effort regulation (Cordovani et al., 2024), and motivational beliefs (Gbollie and Keamu, 2017). Empirical evidence using the MSLQ has consistently linked SRL to academic achievement and persistence (Kurniasari, 2018). Students who actively regulate their learning tend to demonstrate deeper processing strategies (Young, 2005), more effective time management (Wolters and Brady, 2021), and stronger commitment to academic goals (Rahmelia et al., 2025).

Although the MSLQ conceptualizes SRL as multidimensional, the present study focused on overall regulatory competence as a higher-order capacity. Representative items spanning goal setting, monitoring, strategy adjustment, effort regulation, and integrative processing were selected to model SRL as a unidimensional latent construct. This approach is consistent with prior structural equation modeling research in which SRL is treated as an overarching regulatory competence when the theoretical emphasis concerns its downstream psychological and emotional consequences.

Although SRL is multidimensional, it can also be conceptualized as an integrated regulatory capacity when the research purpose is to examine its overall downstream consequences. In the present study, SRL was modeled as a single latent construct because the focus was not on comparing specific SRL components, but on testing whether students' general capacity to regulate cognition, motivation, effort, and strategy use was associated with achievement emotions and academic engagement. Representative items spanning goal setting, monitoring, strategy adjustment, effort regulation, and integrative processing were therefore selected to model SRL as an overall latent construct. This operationalization is consistent with SRL theory, which views self-regulated learners as actively coordinating cognitive, metacognitive, motivational, and behavioral processes during learning. Modeling SRL as an overall latent construct also allowed the present study to reduce model complexity and avoid overparameterization given the sample size.

#### 1.2.2 Self-regulated learning as an antecedent of achievement emotions

Despite the substantial literature linking SRL to performance outcomes, relatively limited research has examined how SRL shapes students' emotional experiences. Theoretically, SRL may influence emotions through its impact on perceived control. Effective planning and monitoring increase predictability in academic tasks, thereby reducing uncertainty-related anxiety. Similarly, successful strategy use can enhance perceived competence and foster enjoyment or hope. Thus, SRL may serve as an antecedent not only of cognitive outcomes but also of emotional experiences within learning contexts.

From the perspective of control–value theory, achievement emotions are shaped by students' appraisals of control and value in achievement-related situations. SRL may contribute to these appraisals because students who set goals, plan strategically, monitor their progress, and regulate effort are more likely to perceive academic tasks as manageable and controllable. Such perceived control can foster positive achievement emotions, including enjoyment and hope, while reducing negative emotions such as anxiety and boredom. Prior research has also suggested that regulatory competence and emotional experience are closely intertwined in academic contexts, as students' use of learning strategies, monitoring processes, and motivational regulation can influence how they emotionally experience learning tasks. Therefore, SRL can be conceptualized not only as a predictor of academic performance, but also as a psychological antecedent of achievement emotions.

### 1.3 Achievement emotions and academic engagement

Achievement emotions, defined as emotions directly linked to achievement activities or outcomes (Pekrun et al., 2011), constitute a critical yet often overlooked dimension of the learning process (Mega et al., 2014). According to control–value theory, achievement emotions arise from students' subjective appraisals of control over learning activities (Pekrun, 2006) and the perceived value of those activities (Zimmermann et al., 2021). Positive achievement emotions such as enjoyment and hope are theorized to broaden cognitive resources (Pekrun et al., 2002a), enhance motivation (Pekrun et al., 2002b), and promote sustained effort (Gao, 2025). Negative emotions such as anxiety and boredom, in contrast, may consume cognitive capacity (Tobias, 1985), reduce persistence (England et al., 2017), and undermine engagement (Dewaele and Li, 2021). Achievement emotions are therefore positioned as proximal determinants of engagement-related behaviors (Berhenke et al., 2011). Nevertheless, existing research tends to examine emotions either as antecedents (Derakhshan and Yin, 2025) or outcomes of engagement (Clark et al., 2014), without systematically integrating them into a broader process model linking regulatory competencies to engagement (Garland et al., 2015).

Achievement emotions are central components of students' academic lives. In the present study, emotions were assessed using the short version of the Achievement Emotions Questionnaire (AEQ-S), which distinguishes between positive emotions (e.g., enjoyment, hope) and negative emotions (e.g., anxiety, boredom) experienced during learning activities. Consistent with control–value theory, these emotional dimensions were modeled separately to examine whether positive and negative pathways operate symmetrically or asymmetrically in predicting engagement.

While both SRL and achievement emotions have independently been linked to engagement, their combined explanatory power remains underexplored. Existing research often examines either direct links between SRL and engagement or direct links between emotions and engagement. Few studies have tested integrative models in which emotions function as mediators between regulatory competencies and engagement outcomes.

#### 1.3.1 Academic engagement as an emotionally embedded process

Academic engagement, typically assessed through behavioral, cognitive, and emotional dimensions, reflects students' sustained involvement in learning. In the present study, academic engagement was measured using four items from the Student Engagement Scale (SES), capturing students' effort investment, persistence, attentional focus, and affective commitment to academic activities. Engagement was conceptualized as an outcome variable reflecting active learning involvement rather than regulatory strategy use *per se*, thereby preserving conceptual distinction between SRL and engagement.

### 1.4 Linking self-regulated learning, achievement emotions, and engagement

The present study seeks to address this gap by proposing and testing a latent mediation model in which achievement emotions mediate the relationship between self-regulated learning and academic engagement among university students. Existing research rarely disentangles the dual emotional pathways within a unified latent framework, leaving unclear whether positive and negative emotions operate symmetrically or asymmetrically. By integrating SRL theory with control–value theory of achievement emotions, this research advances a more comprehensive understanding of how regulatory processes translate into sustained engagement through emotional pathways (Berweger et al., 2025).

Although SRL, achievement emotions, and engagement have each been extensively studied, their interrelations have often been examined in separate research traditions. Studies on SRL have primarily emphasized cognitive strategy use, metacognitive monitoring, persistence, and academic achievement, whereas studies on achievement emotions have often focused on how enjoyment, hope, anxiety, and boredom influence motivation and learning outcomes. A growing body of research suggests that emotions may explain why some students remain behaviorally and cognitively engaged, whereas others withdraw from academic tasks. However, relatively few studies have tested whether positive and negative achievement emotions simultaneously mediate the association between SRL and academic engagement within a unified latent variable framework. This leaves unclear whether the emotional mechanisms linking SRL to engagement operate mainly through the enhancement of positive emotions, the reduction of negative emotions, or both pathways simultaneously.

Integrating self-regulated learning (SRL) theory with control–value theory provides a coherent framework for understanding how regulatory processes influence academic engagement through emotional mechanisms. SRL theory emphasizes that learners actively regulate their cognition, motivation, and behavior through goal setting, strategic planning, self-monitoring, effort regulation, and adaptive strategy use (Pekrun et al., 2002a). These processes may enhance students' perceived control over academic tasks by making learning activities more predictable and manageable (Pintrich, 2000; Zimmerman, 2002). Control–value theory further proposes that achievement emotions arise from students' appraisals of control and value in achievement-related situations (Pekrun, 2006). Thus, students with stronger SRL are likely to experience more positive emotions, such as enjoyment and hope, because they perceive greater control over learning demands, while experiencing fewer negative emotions, such as anxiety and boredom, because uncertainty and perceived uncontrollability are reduced.

Achievement emotions may then shape academic engagement. Positive emotions such as enjoyment and hope are associated with motivation, self-regulated learning, and adaptive academic functioning, thereby facilitating students' behavioral, cognitive, and emotional engagement (Pekrun et al., 2002a; Mega et al., 2014). Negative emotions such as anxiety and boredom may consume cognitive resources, weaken persistence, and promote disengagement or avoidance behaviors, thereby undermining engagement (Tobias, 1985; Dewaele and Li, 2021). Therefore, achievement emotions can be conceptualized as proximal mediators linking SRL as a distal regulatory competence to students' observable engagement behaviors. On this basis, the present study examines whether positive and negative achievement emotions mediate the relationship between SRL and academic engagement.

### 1.5 Research questions and hypotheses

Based on the theoretical integration outlined above, the present study addresses the following research questions:

Does self-regulated learning positively predict academic engagement among university students?

Does self-regulated learning positively predict positive achievement emotions and negatively predict negative achievement emotions?

Do positive and negative achievement emotions mediate the relationship between self-regulated learning and academic engagement?

Are the indirect effects through positive and negative emotions comparable in magnitude?

The following hypotheses are proposed:

H1: Self-regulated learning is positively associated with academic engagement.

H2a: Self-regulated learning is positively associated with positive achievement emotions.

H2b: Self-regulated learning is negatively associated with negative achievement emotions.

H3a: Positive achievement emotions are positively associated with academic engagement.

H3b: Negative achievement emotions are negatively associated with academic engagement.

H4: Positive and negative achievement emotions mediate the relationship between self-regulated learning and academic engagement.

## 2 Method

### 2.1 Participants

Participants were initially 230 undergraduate students recruited from a comprehensive university in China. After data screening, 207 valid cases were retained for analysis. All participants in the final sample were full-time students enrolled in at least one academic course during the data collection period. The sample included students from multiple academic disciplines, including social sciences, natural sciences, and humanities, ensuring disciplinary diversity.

The final sample size of 207 was considered acceptable for the specified latent SEM model, which included four latent constructs with a limited number of indicators. Model convergence was achieved without estimation problems, and all standard errors were within acceptable ranges, suggesting that the sample was adequate for the planned analyses.

Participation was voluntary, and no course credit or monetary compensation was provided. The study protocol was reviewed and approved by the institutional ethics committee of the authors' university.

### 2.2 Procedure

Data were collected during a regular academic semester using an online questionnaire platform. Prior to participation, students received written information describing the purpose of the study, the voluntary nature of participation, and their right to discontinue at any time without consequences. Electronic informed consent was obtained before the survey commenced.

Participants completed a battery of self-report questionnaires assessing self-regulated learning, achievement emotions, and academic engagement. The average completion time was approximately 15–20 min. To reduce common method bias, all responses were collected anonymously, no identifying information was recorded, and scale items were presented in their original validated formats.

### 2.3 Measures

All measures were administered in Chinese using previously validated translations. Responses across all instruments were recorded on five-point Likert-type scales, with higher scores indicating higher levels of the corresponding construct.

#### 2.3.1 Self-regulated learning

Self-regulated learning (SRL) was assessed using selected items from the Motivated Strategies for Learning Questionnaire (MSLQ). Eight items were used to capture core regulatory components, including cognitive strategy use, metacognitive regulation, effort regulation, and goal-directed behavior. These items correspond to indicators commonly employed to represent SRL as a higher-order latent construct in SEM research.

Participants responded on a scale ranging from 1 (not at all true of me) to 5 (very true of me). In the present sample, the SRL scale demonstrated acceptable internal consistency (Cronbach's α = 0.80), meeting recommended reliability thresholds. Confirmatory factor analysis supported a unidimensional latent SRL construct, with all indicators showing substantial and statistically significant factor loadings.

#### 2.3.2 Achievement emotions

Achievement emotions were measured using the short version of the Achievement Emotions Questionnaire (AEQ-S). Consistent with control–value theory, achievement emotions were modeled as two higher-order latent variables: positive achievement emotions and negative achievement emotions.

Positive achievement emotions were assessed using four items representing enjoyment and hope, while negative achievement emotions were assessed using five items representing anxiety and boredom. Participants rated each item from 1 (strongly disagree) to 5 (strongly agree).

Internal consistency was good for both dimensions (α = 0.86 for positive emotions; α = 0.88 for negative emotions). Confirmatory factor analysis indicated that observed indicators loaded significantly on their respective latent factors, supporting the factorial validity of the two-dimensional emotion structure.

#### 2.3.3 Academic engagement

Academic engagement was assessed using four items from the Student Engagement Scale (SES), capturing students' behavioral persistence, cognitive involvement, and emotional commitment to academic learning activities. Responses ranged from 1 (strongly disagree) to 5 (strongly agree). Higher scores indicated stronger engagement.

The engagement scale demonstrated good reliability in the present sample (Cronbach's α = 0.90). Confirmatory factor analysis supported a unidimensional latent engagement construct, with all items loading strongly on the latent factor.

Descriptive statistics, reliability coefficients, and zero-order correlations based on observed composite scores are reported in Table 1.

**Table 1:** Means, standard deviations, Cronbach's α, and correlations among study variables (*N* = 207).

Variable	*M*	SD	α	1	2	3	4
1. Self-regulated learning (SRL)	2.93	1.04	0.80	–			
2. Positive achievement emotions	2.99	1.02	0.86	0.48^***^	–		
3. Negative achievement emotions	3.02	1.03	0.88	−0.46^***^	−0.16	–	
4. Academic engagement	2.94	1.05	0.90	0.60^***^	0.47^***^	−0.49^***^	–

^***^p < 0.001.

Correlations are based on observed scale scores.

### 2.4 Data analysis

Data were analyzed using Mplus Version 8.3. Structural equation modeling (SEM) with latent variables was employed to test the hypothesized parallel mediation model. The robust maximum likelihood estimator (MLR) was used to account for potential deviations from multivariate normality.

Analyses followed a two-step approach. A measurement model was first estimated to evaluate the factorial validity of all latent constructs, including self-regulated learning, positive achievement emotions, negative achievement emotions, and academic engagement. Model fit was assessed using multiple indices: comparative fit index (CFI), Tucker–Lewis index (TLI), root mean square error of approximation (RMSEA), and standardized root mean square residual (SRMR). Conventional cutoff criteria were applied (CFI/TLI ≥ 0.90; RMSEA ≤ 0.08; SRMR ≤ 0.08).

After establishing an acceptable measurement model, a structural model was specified to test the hypothesized relationships. Self-regulated learning was modeled as the exogenous variable, academic engagement as the endogenous outcome, and positive and negative achievement emotions as parallel mediators. Direct, indirect, and total effects were estimated simultaneously.

Indirect effects were calculated using the delta method, and statistical significance was evaluated based on *z* values and 95% confidence intervals. Standardized coefficients (STDYX) are reported to facilitate interpretation. Structural path estimates and mediation effects are presented in Table 2.

**Table 2:** Standardized direct, indirect, and total effects from the structural equation model.

Path	β (STDYX)	SE	*z*	*p*
SRL → positive emotions	0.484	0.075	6.47	<0.001
SRL → negative emotions	−0.456	0.067	−6.78	<0.001
Positive emotions → engagement	0.262	0.081	3.25	0.001
Negative emotions → engagement	−0.297	0.077	−3.87	<0.001
SRL → engagement (direct)	0.332	0.089	3.75	<0.001
Total effect	0.595	0.051	11.68	<0.001
Total indirect effect	0.263	0.063	4.16	<0.001
Indirect via positive emotions	0.127	0.046	2.76	0.006
Indirect via negative emotions	0.136	0.040	3.41	0.001

SRL, self-regulated learning.

Standardized estimates are reported using the STDYX metric.

Model fit: χ^2^(183) = 200.48, *p* = 0.179, CFI = 0.991, TLI = 0.989, RMSEA = 0.021, SRMR = 0.040.

To examine potential common method bias arising from the use of self-report measures collected at a single time point, Harman's single-factor test and a single-factor CFA comparison were conducted. Harman's single-factor test showed that the first unrotated factor accounted for 29.84% of the total variance, which was below the commonly used threshold of 40%. In addition, a single-factor CFA model, in which all observed items were constrained to load onto one latent factor, showed substantially poorer fit than the proposed four-factor measurement model (ΔCFI > 0.10; ΔRMSEA > 0.05). These analyses provided preliminary evidence that a single general factor did not dominate the covariance structure. However, these procedures are limited and cannot fully rule out common method variance. Therefore, the findings should be interpreted with caution. Future studies should employ more robust approaches, such as latent method factor models, multi-informant data, behavioral indicators, or longitudinal designs.

### 2.5 Ethical considerations

The study was conducted in accordance with the ethical principles outlined in the Declaration of Helsinki. Ethical approval was obtained from the institutional ethics committee of the affiliated university prior to data collection. All participants provided informed consent electronically. Data were collected anonymously and used exclusively for research purposes.

## 3 Result

### 3.1 Preliminary analyses

**Figure 1:**
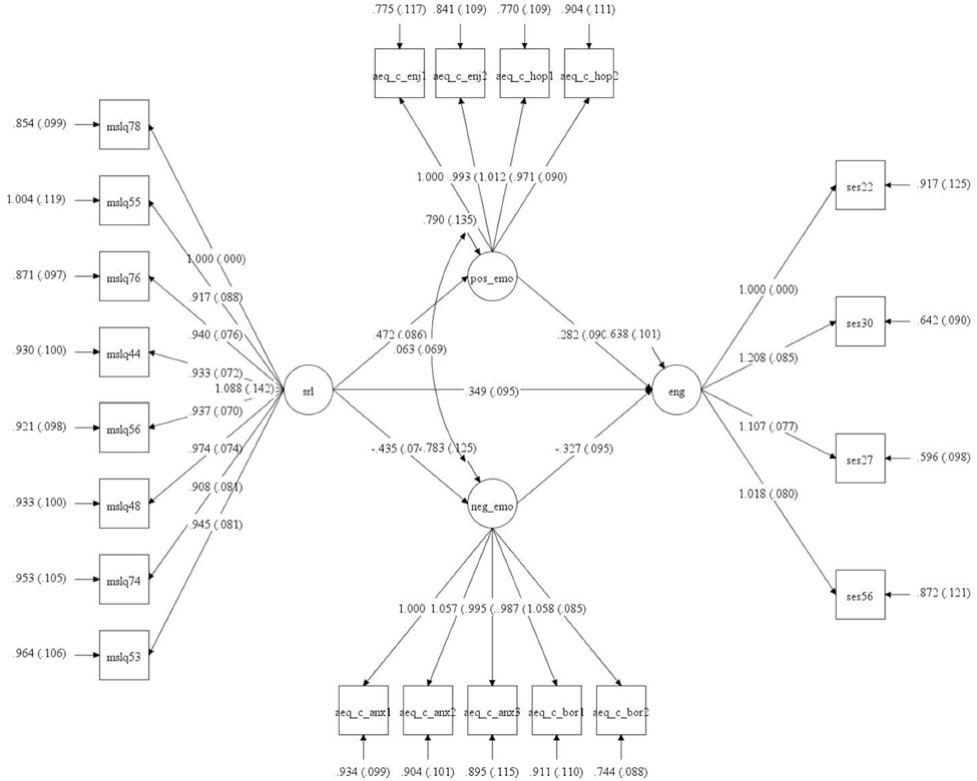
Structural equation model of self-regulated learning, achievement emotions, and academic engagement.

Descriptive statistics, internal consistency coefficients, and zero-order correlations among the study variables are presented in Table 1. All measures demonstrated acceptable to excellent reliability, with Cronbach's α values ranging from 0.798 to 0.90. Specifically, the reliability coefficient was 0.798 for self-regulated learning, 0.86 for positive achievement emotions, 0.88 for negative achievement emotions, and 0.90 for academic engagement. The structural equation model is presented in Figure 1.

The correlation results showed that self-regulated learning was positively correlated with positive achievement emotions and academic engagement, and negatively correlated with negative achievement emotions. Positive achievement emotions were positively associated with academic engagement, whereas negative achievement emotions were negatively associated with academic engagement. These preliminary results were consistent with the proposed theoretical model and provided initial support for the hypothesized associations among self-regulated learning, achievement emotions, and academic engagement.

### 3.2 RQ1/H1: relationship between self-regulated learning and academic engagement

RQ1 examined whether self-regulated learning was positively associated with academic engagement among university students. H1 proposed that self-regulated learning would be positively associated with academic engagement.

The structural equation modeling results supported H1. The total effect of self-regulated learning on academic engagement was significant and positive, β = 0.595, SE = 0.051, *z* = 11.68, *p* < 0.001. After accounting for the mediating effects of positive and negative achievement emotions, the direct path from self-regulated learning to academic engagement remained statistically significant, β = 0.332, SE = 0.089, *z* = 3.75, *p* < 0.001.

These findings indicate that students with stronger self-regulated learning were more likely to show higher levels of academic engagement. The significant direct effect also suggests that self-regulated learning contributes to engagement not only through emotional mechanisms but also through other regulatory processes, such as goal setting, strategy use, monitoring, and effort regulation.

These results supported H1, indicating that SRL was positively associated with academic engagement. The significant direct effect after accounting for achievement emotions further suggests that SRL contributed to engagement beyond the two emotional mediating pathways.

### 3.3 RQ2/H2: relationship between self-regulated learning and achievement emotions

RQ2 examined whether self-regulated learning predicted positive and negative achievement emotions. H2a proposed that self-regulated learning would be positively associated with positive achievement emotions, whereas H2b proposed that self-regulated learning would be negatively associated with negative achievement emotions.

The results supported both H2a and H2b. Self-regulated learning significantly and positively predicted positive achievement emotions, β = 0.484, SE = 0.075, *z* = 6.47, *p* < 0.001. This result indicates that students with stronger self-regulated learning tended to report higher levels of positive achievement emotions, such as enjoyment and hope.

Self-regulated learning also significantly and negatively predicted negative achievement emotions, β = −0.456, SE = 0.067, *z* = −6.78, *p* < 0.001. This finding indicates that students with stronger self-regulated learning tended to report lower levels of negative achievement emotions, such as anxiety and boredom.

Together, these results suggest that self-regulated learning is not only related to students' behavioral and cognitive involvement in learning, but also to their emotional experiences in academic contexts.

These findings supported H2a and H2b, showing that SRL was positively associated with positive achievement emotions and negatively associated with negative achievement emotions.

### 3.4 RQ3/H3–H4: mediating effects of positive and negative achievement emotions

RQ3 examined whether positive and negative achievement emotions mediated the relationship between self-regulated learning and academic engagement. H3a proposed that positive achievement emotions would be positively associated with academic engagement, and H3b proposed that negative achievement emotions would be negatively associated with academic engagement. H4 proposed that achievement emotions would mediate the relationship between self-regulated learning and academic engagement.

The SEM results supported H3a and H3b. Positive achievement emotions were significantly and positively associated with academic engagement, β = 0.262, SE = 0.081, *z* = 3.25, *p* = 0.001. This result suggests that students who experienced more enjoyment and hope were more likely to invest effort, persist in learning tasks, and remain actively engaged in academic activities.

Negative achievement emotions were significantly and negatively associated with academic engagement, β = −0.297, SE = 0.077, *z* = −3.87, *p* < 0.001. This result suggests that students who experienced more anxiety and boredom were less likely to maintain academic engagement.

The mediation analysis further supported H4. The total indirect effect of self-regulated learning on academic engagement through achievement emotions was significant, β = 0.263, SE = 0.063, *z* = 4.16, *p* < 0.001. The specific indirect effect through positive achievement emotions was significant, β = 0.127, SE = 0.046, *z* = 2.76, *p* = 0.006. The specific indirect effect through negative achievement emotions was also significant, β = 0.136, SE = 0.040, *z* = 3.41, *p* = 0.001.

These findings indicate that both positive and negative achievement emotions functioned as significant mediators between self-regulated learning and academic engagement. In other words, self-regulated learning promoted academic engagement partly by increasing positive achievement emotions and partly by reducing negative achievement emotions.

These results supported H3a, H3b, and H4, indicating that positive and negative achievement emotions were both significantly associated with academic engagement and served as parallel mediators between SRL and engagement.

### 3.5 RQ4: comparison of the two emotional pathways

RQ4 examined whether the indirect effects through positive and negative achievement emotions were comparable in magnitude. The mediation results showed that both emotional pathways were statistically significant. The indirect effect through positive achievement emotions was β = 0.127, whereas the indirect effect through negative achievement emotions was β = 0.136.

The two indirect effects were similar in size, suggesting that positive and negative achievement emotions made relatively comparable contributions to the relationship between self-regulated learning and academic engagement. The total indirect effect was β = 0.263, and the total effect was β = 0.595. Therefore, the proportion mediated was calculated as 0.263/0.595 = 0.442, indicating that approximately 44.2% of the total effect of self-regulated learning on academic engagement operated through achievement emotions.

This result suggests that the emotional mechanism linking self-regulated learning to academic engagement is bidirectional in nature: self-regulated learning enhances engagement both by fostering adaptive emotions, such as enjoyment and hope, and by reducing maladaptive emotions, such as anxiety and boredom. The comparable magnitude of the two pathways highlights the importance of considering both positive and negative achievement emotions when explaining how self-regulated learning contributes to academic engagement.

## 4 Discussion

The present study examined the relationship between self-regulated learning (SRL) and academic engagement among university students by testing a latent parallel mediation model of achievement emotions. Drawing on SRL theory and control–value theory, the study investigated whether positive and negative achievement emotions mediated the association between SRL and academic engagement. The findings supported the proposed model. SRL was positively associated with positive achievement emotions and academic engagement, and negatively associated with negative achievement emotions. Both positive and negative achievement emotions significantly predicted academic engagement and served as parallel mediators. These findings suggest that SRL contributes to academic engagement not only through direct regulatory processes, but also through students' emotional experiences during academic learning.

### 4.1 SRL and achievement emotions

#### 4.1.1 SRL as a source of perceived control

The finding that SRL significantly predicted both positive and negative achievement emotions provides support for the view that regulatory competence is closely related to students' emotional experiences. SRL theory conceptualizes learners as active agents who set goals, select strategies, monitor progress, regulate effort, and adjust learning behaviors in response to academic demands (Pintrich, 2000; Zimmerman, 2002). These regulatory processes may enhance students' perceived control over learning tasks because students who plan effectively and monitor their progress are more likely to view academic tasks as manageable and predictable.

Control–value theory provides a useful explanation for these findings. According to this theory, achievement emotions arise from students' appraisals of control and value in achievement-related situations (Pekrun, 2006; Pekrun et al., 2011). The positive association between SRL and positive achievement emotions suggests that students who regulate their learning effectively may experience greater perceived control over academic tasks. Goal setting, planning, monitoring, and strategy adjustment can make learning activities more predictable and manageable, thereby increasing enjoyment and hope. Conversely, the negative association between SRL and negative achievement emotions suggests that regulatory competence may reduce uncertainty, helplessness, and task aversion, which are closely related to anxiety and boredom. The significant indirect effects further indicate that these emotional experiences are not peripheral outcomes, but central mechanisms through which SRL contributes to academic engagement.

This result also extends previous research by showing that SRL is not only associated with academic achievement, persistence, or strategy use, but also with achievement emotions. Previous studies have suggested that emotions, motivation, and self-regulated learning are closely interconnected in academic contexts (Mega et al., 2014). The present study adds to this literature by demonstrating, at the latent construct level, that students with stronger SRL reported higher positive achievement emotions and lower negative achievement emotions.

#### 4.1.2 Differentiated effects on positive and negative emotions

The results showed that SRL was positively associated with positive achievement emotions and negatively associated with negative achievement emotions. These differentiated effects are theoretically meaningful. Positive achievement emotions, such as enjoyment and hope, may arise when students perceive that they can successfully manage academic tasks and make progress toward learning goals. SRL strategies such as planning, monitoring, and effort regulation may therefore increase positive emotional experiences by strengthening students' sense of competence and control.

Negative achievement emotions, such as anxiety and boredom, may be reduced when students are able to regulate their learning effectively. Anxiety is often associated with uncertainty and perceived inability to meet academic demands, whereas boredom may emerge when students experience low task involvement or low perceived value. Students with stronger SRL may be better able to clarify task requirements, sustain effort, and adjust strategies, which can reduce feelings of uncontrollability and disengagement. This finding is consistent with the control–value perspective that different emotions emerge from different combinations of control and value appraisals.

The differentiated associations also support the decision to model positive and negative achievement emotions as separate latent constructs. Positive and negative emotions should not be treated simply as opposite ends of a single emotional continuum. Rather, they may reflect related but distinct emotional processes that have different antecedents and consequences in academic learning.

### 4.2 Achievement emotions and academic engagement

#### 4.2.1 Positive emotional pathway

Positive achievement emotions were significantly and positively associated with academic engagement. This finding suggests that students who experience more enjoyment and hope are more likely to invest effort, maintain attention, persist in academic tasks, and participate actively in learning activities. Positive emotions may energize students' learning behavior by increasing intrinsic motivation, broadening cognitive resources, and supporting sustained involvement.

This result is consistent with previous research showing that positive academic emotions contribute to adaptive learning processes and academic outcomes (Pekrun et al., 2002b; Mega et al., 2014). From the perspective of control–value theory, enjoyment and hope are not merely pleasant emotional states; they are motivationally significant experiences that can promote students' willingness to engage with academic tasks. In this sense, positive achievement emotions may serve as psychological resources that help translate students' regulatory competence into active academic engagement.

The positive emotional pathway also has practical significance. If students' enjoyment and hope can be strengthened through effective instructional design, autonomy support, constructive feedback, and opportunities for successful strategy use, their engagement may increase. Thus, promoting SRL and cultivating positive achievement emotions may be mutually reinforcing approaches to enhancing academic engagement.

#### 4.2.2 Negative emotional pathway

Negative achievement emotions were significantly and negatively associated with academic engagement. This finding indicates that students who experience more anxiety and boredom are less likely to remain behaviorally, cognitively, and emotionally involved in academic learning. Anxiety may consume cognitive resources and interfere with attention, whereas boredom may reduce motivation, persistence, and task involvement. Both emotions can therefore undermine engagement.

This finding is consistent with earlier research showing that anxiety can interfere with learning processes by occupying cognitive capacity (Tobias, 1985). It is also consistent with studies showing that boredom is closely related to disengagement and reduced learning involvement. For example, Dewaele and Li (2021) found that enjoyment and boredom played important roles in shaping students' learning engagement in classroom contexts. The present study extends this evidence by showing that negative achievement emotions function as a significant pathway linking SRL to academic engagement.

The negative emotional pathway suggests that improving engagement requires more than encouraging students to use learning strategies. It is also necessary to reduce emotional barriers that prevent students from sustaining academic involvement. Reducing excessive uncertainty, clarifying academic expectations, increasing task value, and providing timely feedback may help lower anxiety and boredom, thereby supporting engagement.

### 4.3 Mediation effects and theoretical interpretation

#### 4.3.1 Partial mediation

The mediation results showed that achievement emotions partially mediated the relationship between SRL and academic engagement. The total indirect effect was significant, and approximately 44.2% of the total effect of SRL on engagement operated through achievement emotions. This finding indicates that emotional experiences explain a substantial part of the SRL–engagement relationship.

At the same time, the direct effect of SRL on academic engagement remained significant after accounting for positive and negative achievement emotions. This suggests that achievement emotions are important but not exhaustive mechanisms. SRL may also promote engagement through other pathways, such as time management, metacognitive monitoring, strategic persistence, task planning, and effort regulation. Students with stronger SRL may be more engaged because they know how to organize their learning, monitor their progress, and sustain effort even when academic tasks are difficult.

This partial mediation pattern is theoretically important because it suggests that the SRL–engagement relationship should not be understood as either purely cognitive-regulatory or purely emotional. Instead, academic engagement may be associated with both regulatory competence and emotional experience. SRL may be linked to students' engagement through strategic learning behaviors, while also being indirectly associated with engagement through higher positive emotions and lower negative emotions. Although the mediation model was theoretically grounded, the cross-sectional and correlational nature of the data means that the findings should be interpreted as evidence of statistical mediation rather than causal mediation. Reciprocal relations may also exist among SRL, achievement emotions, and academic engagement.

#### 4.3.2 Balanced positive and negative emotional pathways

Both positive and negative achievement emotions served as significant mediators. The indirect effect through positive achievement emotions and the indirect effect through negative achievement emotions were similar in magnitude. This pattern suggests that the emotional mechanism linking SRL to academic engagement operates through two complementary pathways: enhancing positive emotions and reducing negative emotions.

The positive pathway indicates that SRL may increase academic engagement by fostering enjoyment and hope. The negative pathway indicates that SRL may also increase engagement by reducing anxiety and boredom. These findings are consistent with control–value theory, which emphasizes that different achievement emotions may have distinct antecedents and consequences depending on students' control and value appraisals.

This balanced pattern has important implications. Interventions that focus only on increasing positive emotions may overlook the disengaging effects of anxiety and boredom. Similarly, interventions that focus only on reducing negative emotions may fail to cultivate the motivational benefits of enjoyment and hope. Therefore, a comprehensive approach to academic engagement should address both sides of students' emotional experience.

### 4.4 Theoretical and methodological contributions

The present study makes several theoretical and methodological contributions. Theoretically, it integrates SRL theory with control–value theory to explain how students' regulatory competence is linked to academic engagement through achievement emotions. Previous research has often examined SRL, emotions, and engagement separately. The present study brings these constructs together in a unified model and shows that achievement emotions are important mechanisms through which SRL contributes to engagement.

The study also contributes to the literature by distinguishing between positive and negative achievement emotions. Rather than treating emotional experience as a single general affective factor, the model tested positive and negative emotions as parallel mediators. The findings show that both emotional dimensions independently contributed to academic engagement. This supports the view that positive and negative achievement emotions are related but distinct processes.

Methodologically, the study used latent variable modeling to test the hypothesized relationships. This approach reduces measurement error and allows for a more precise estimation of the associations among SRL, achievement emotions, and academic engagement. By using a latent parallel mediation model, the study provides stronger evidence for the emotional mechanisms linking SRL and engagement than would be available from observed composite-score analyses alone.

### 4.5 Practical implications

The findings offer several implications for higher education practice. First, promoting SRL should remain a central goal of teaching and student development. Instructors can support SRL by helping students set clear learning goals, plan study strategies, monitor progress, regulate effort, and reflect on learning outcomes. Such practices may directly enhance engagement and indirectly promote engagement through improved emotional experiences.

Second, emotional processes should be explicitly considered in engagement-oriented interventions. Because positive achievement emotions were associated with higher engagement, instructors should create learning environments that foster enjoyment and hope. This can be achieved through meaningful learning tasks, autonomy-supportive teaching, constructive feedback, and opportunities for students to experience progress and competence.

Third, reducing negative achievement emotions is also important. Anxiety and boredom can undermine engagement, so instructors should reduce unnecessary ambiguity, clarify task expectations, provide appropriate challenge levels, and increase the perceived value of academic tasks. Students may also benefit from support in coping with academic stress, managing uncertainty, and maintaining attention during difficult or repetitive learning activities.

Finally, the findings suggest that SRL training and emotion-supportive instruction should be integrated. Teaching students learning strategies without addressing their emotional experiences may limit the effectiveness of engagement interventions. Similarly, promoting positive emotions without strengthening regulatory skills may not be sufficient for sustained engagement. A combined approach may be more effective in supporting university students' academic development.

### 4.6 Limitations and future research

The study used a cross-sectional design, which limits causal interpretation. Therefore, the mediation results should be interpreted as statistical mediation rather than causal mediation. Longitudinal or experimental studies are needed to examine the temporal and causal dynamics among SRL, achievement emotions, and academic engagement. Several limitations should be considered. First, the study used a cross-sectional design, which limits causal interpretation. Although the proposed model was theoretically grounded, the temporal ordering among SRL, achievement emotions, and academic engagement cannot be firmly established. Future studies should use longitudinal or experimental designs to examine how SRL, emotions, and engagement influence one another over time.

Second, the sample was limited to Chinese undergraduates from one institutional and cultural context, which restricts the generalizability of the findings. Students' SRL, achievement emotions, and academic engagement may be shaped by cultural expectations, instructional practices, assessment systems, and institutional environments. Therefore, the present findings should be generalized with caution. Future research should test the proposed model across different countries, universities, academic disciplines, and educational levels to examine whether the emotional mediation pathways identified in this study are culturally and contextually robust.

Third, the sample was drawn from university students in one institutional and cultural context. Future research should examine whether the proposed model holds across different universities, academic disciplines, cultural settings, and educational levels. Such studies would help determine the generalizability of the emotional mediation model.

Fourth, the present study focused on positive and negative achievement emotions as broad emotional categories. Future research could examine specific emotions separately, such as enjoyment, hope, anxiety, and boredom, to determine whether each emotion plays a distinct mediating role. It would also be useful to include direct measures of perceived control and task value, which are central constructs in control–value theory, to test the appraisal mechanisms more explicitly.

Finally, future studies could examine possible moderators of the mediation model, such as gender, academic discipline, instructional support, perceived autonomy, or academic stress. These factors may influence the strength of the relationships among SRL, achievement emotions, and academic engagement.

### 4.7 Conclusion

This study provides empirical support for a latent mediation model in which achievement emotions partially mediate the relationship between self-regulated learning and academic engagement. SRL was associated with higher positive achievement emotions and lower negative achievement emotions, both of which contributed to academic engagement. The findings highlight achievement emotions as important psychological mechanisms linking regulatory competence to sustained academic involvement. Overall, the study suggests that enhancing university students' engagement requires attention to both self-regulated learning processes and the emotional experiences that accompany academic learning.

## Data Availability

The raw data supporting the conclusions of this article will be made available by the authors, without undue reservation.
