# Supplementary material for: Self-regulated learning and academic engagement among university students: a latent mediation model of achievement emotions
Source: Front Psychol. 2026 Jun 11;17:1822649. doi: 10.3389/fpsyg.2026.1822649 (PMC13294044; doi:10.3389/fpsyg.2026.1822649)
Supplement: Supplementary file 1 [file Supplementary_file_1.pdf]

Appendix A. Questionnaire Items Used in the Present Study

All questionnaire items used in the present study are listed below. The items were grouped according to their corresponding scales and dimensions. Participants responded to all items on a five-point Likert-type scale, with higher scores indicating higher levels of the corresponding construct.

Table A1. Summary of scales used in the present study

Scale	Dimension	Number of items	Cronbach’s α
Self-Regulated Learning	Planning/goal setting, metacognitive monitoring, regulation/strategy use, effort regulation, reflection/integration	8	.798
Positive Achievement Emotions	Enjoyment, hope/confidence	4	.86
Negative Achievement Emotions	Anxiety, boredom	5	.88
Academic Engagement	Cognitive engagement, behavioral engagement	4	.90

Table A2. Questionnaire items used in the present study

Scale	Dimension	Item no.	Item wording
Self-Regulated Learning	Planning / Goal setting	SRL1	When I study for this class, I set goals for myself in order to direct my activities in each study period.
Self-Regulated Learning	Metacognitive monitoring	SRL2	I ask myself questions to make sure I understand the material I have been studying in this class.
Self-Regulated Learning	Metacognitive monitoring	SRL3	When studying for this course, I try to determine which concepts I don' t understand well.
Self-Regulated Learning	Regulation / Strategy use	SRL4	If course readings are difficult to understand, I change the way I read the material.
Self-Regulated Learning	Regulation / Strategy use	SRL5	I try to change the way I study in order to fit the course requirements and the instructor' s teaching style.
Self-Regulated Learning	Effort regulation	SRL6	I work hard to do well in this class even if I don' t like what we are doing.
Self-Regulated Learning	Effort regulation	SRL7	Even when course materials are dull and uninteresting, I manage to keep working until I finish.
Self-Regulated Learning	Reflection / Integration	SRL8	When I study for this class, I pull together information from different sources, such as lectures, readings, and discussions.
Positive Achievement Emotions	Enjoyment	PAE1	I enjoy being in class.
Positive Achievement Emotions	Enjoyment	PAE2	I am motivated to go to this class because it' s exciting.
Positive Achievement	Hope / Confidence	PAE3	I am confident when I go to class.

Scale	Dimension	Item no.	Item wording
Emotions			
Positive Achievement Emotions	Hope / Confidence	PAE4	Being confident that I will understand the material motivates me.
Negative Achievement Emotions	Anxiety	NAE1	I feel nervous in class.
Negative Achievement Emotions	Anxiety	NAE2	Even before class, I worry whether I will be able to understand the material.
Negative Achievement Emotions	Anxiety	NAE3	I get tense in class.
Negative Achievement Emotions	Boredom	NAE4	I get bored.
Negative Achievement Emotions	Boredom	NAE5	I think about what else I might be doing rather than sitting in this boring class.
Academic Engagement	Cognitive engagement	AE1	I determine my own learning goals.
Academic Engagement	Cognitive engagement	AE2	I spend enough time and make enough effort to learn.
Academic Engagement	Behavioral engagement	AE3	I attend classes by getting prepared in advance.
Academic Engagement	Behavioral engagement	AE4	I do my homework/tasks in time.
